# Ultraflexible and Mechanically Strong Polymer/Polyaniline Conductive Interpenetrating Nanocomposite via In Situ Polymerization of Vinyl Monomer

**DOI:** 10.3390/polym13132159

**Published:** 2021-06-30

**Authors:** Haihua Wang, Xiaojing Wu, Xuan Qin, Guiqiang Fei, Liyu Sun, Yanyu Li, Mengxi Wang

**Affiliations:** Shaanxi Key Laboratory of Chemical Additives for Industry, College of Chemistry and Chemical Engineering, Shaanxi University of Science and Technology, Xi’an 710021, China; 1808042@sust.edu.cn (X.W.); 50851@sust.edu.cn (X.Q.); sunliyu@sust.edu.cn (L.S.); 13630232961@163.com (Y.L.); jasminewang@sust.edu.cn (M.W.)

**Keywords:** polyaniline, polyacrylate, conductive, flexible, mechanical property

## Abstract

Simultaneous enhancement of conductivity and mechanical properties for polyaniline/polymer nanocomposite still remains a big challenge. Here, a reverse approach via in situ polymerization (RIP) of vinyl monomers in waterborne polyaniline dispersion was raised to prepare conductive polyaniline (GPANI)/polyacrylate (PMB) interpenetrating polymer (GPANI-PMB) nanocomposite. GPANI/PMB physical blend was simultaneously prepared as reference. The conductive GPANI-PMB nanocomposite film with compact pomegranate-shape morphology is homogeneous, ultraflexible and mechanically strong. With incorporating a considerable amount of PMB into GPANI via the RIP method, only a slight decrease from 3.21 to 2.80 S/cm was detected for the conductivity of GPANI-PMB, while the tensile strength significantly increased from 25 to 43.5 MPa, and the elongation at break increased from 40% to 234%. The water absorption of GPANI-PMB3 after 72 h immersion decreased from 24.68% to 10.35% in comparison with GPANI, which is also higher than that of GPANI/PMB. The conductivity and tensile strength of GPANI-PMB were also much higher than that of GPANI/PMB (0.006 S/cm vs. 5.59 MPa). Moreover, the conductivity of GPANI-PMB remained almost invariable after folding 200 times, while that of GPANI/PMB decreased by almost half. This RIP approach should be applicable for preparing conventional conductive polymer nanocomposite with high conductivity, high strength and high flexibility.

## 1. Introduction

With the development of 5G technology and artificial intelligence (AI) technology, wearable, portable and stretchable devices will be of significant and widespread utilization, with extensive applications in the fields of motion detection, health monitoring, and artificial intelligence [1]. This trend substantially increases the demand for high-performance and functional elastic materials [2]. Elastic and conductive polymer nanocomposites have been extensively investigated due to their excellent deformation capability compared to the traditional rigid metal and semiconductors [3].

Incorporation of conductive substances such as silver [4,5], graphene [6], liquid metal [7], carbon nanotube [8] and conducting polymers (such as PEDOT:PSS [9] and PANI [10,11,12], etc.), into polymer matrix is a common approach to fabricate stretchable and conductive polymer composite. PANI has obtained increasing attention in recent decades owing to its unique functions such as simple synthesis, having a promising capability in conductivity, photoelectric properties [13], electrocatalytic activity [14], thermal stability [15], remarkable energy storage characteristics [16] and good environmental stability [17]. Therefore, it has been widely used in sensors [18], biosensors [19], biofuel cells [20] and electrochromic devices [21]. However, its poor mechanical stretchability and flexibility restricted its practical application to flexible and stretchable devices [17,22]. In situ polymerization of aniline in polymer matrix [23,24], immersion method [23] and chemical grafting method [16,25] have been adopted to prepare PANI/polymer composite. For instance, Wu et al. prepared conductive PANI/polyacrylate composite through chemical oxidation polymerization of aniline in polyacrylate (PA) emulsion with the assistance of hydrophilic sulfonated stabilizer. The volume resistance and tensile strength of PANI/PA were 9.9 Ω.cm and 2.81 MPa with the addition of 20 wt% PANI [23]. Zhang et al. prepared flexible polyethylene terephthalate/PANI (PET/PANI) composite paper via in situ polymerization of aniline on PET paper by immersing PET paper in aniline solution, and its electrical conductivity reached 0.78 S/cm [24]. Luan et al. have fabricated elastomer/polyaniline composites via dipping polyurethane sponges into PANI solution and thereby obtained conductive elastomer with an electrical conductivity of 2.38 × 10^−4^ S/cm at 72 wt% of PANI content for stretchable conductors [23]. In our previous study, PANI-graft-poly (vinyl alcohol) with good conductivity and tensile strength was fabricated via chemical graft of PANI onto epoxy modified poly (vinyl alcohol) [16]. However, the water resistance was destined to be poor when the pure hydrophilic poly (vinyl alcohol) was mainly used as polymer matrix.

In order to address the aforementioned challenges, i.e., simultaneously improving the electrical conductivity, mechanical property and water resistance of polymer/PANI composite, a facile approach was put forward to prepare polymer/PANI composite. Herein, a reverse in situ polymerization approach (RIP) via in situ polymerization of vinyl monomers in waterborne polyaniline dispersion was put forward to fabricate high-performance conductive polyaniline/polyacrylate interpenetrating nanocomposite (GPANI-PMB) for the first time. Typical soft vinyl monomer butyl acrylate (BA) and hard vinyl monomer methyl methacrylate (MMA) were adopted, and effects of BA/MMA ratio on the morphology and properties of GPANI-PMB were investigated.

## 2. Materials and Methods

### 2.1. Materials

Aniline was supplied by Tianjin Chemical Reagent Factory and purified by double distillation under reduced pressure prior to use. Poly(vinyl alcohol) (PVA: Pn, 0588 ± 50; Mw, 19,800–24,200) was obtained from Shanghai Yingjia Industrial Development Co., Ltd., Shanghai, China. Glycidyl methacrylate (GMA), butyl acrylate (BA), methyl methacrylate cerium ammonium nitrate (CAN), ammonium persulfate (APS), hydrochloric acid (HCl) and sodium lauryl sulfate (SDS) were supplied by Tianjin Tianli Chemical Reagent Co., Ltd., Tianjin, China. Nitric acid was purchased from Sichuan Xilong chemical Co. Ltd., Sichuan, China.

### 2.2. Preparation of GPANI-PMB and GPANI/PMB Dispersions

**Figure 1:**
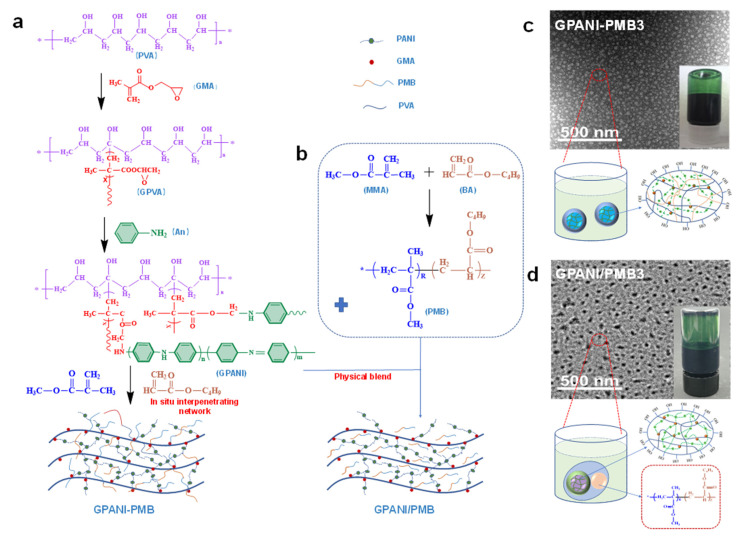
Schematic preparation process of (**a**) GPANI-PMB by the reverse in situ polymerization of vinyl monomers in GPANI and (**b**) GPANI/PMB dispersions by physical blend; TEM images and schematic models of (**c**) GPANI-PMB and (**d**) GPANI/PMB dispersions, the inserted images are optical photographs of GPANI-PMB and GPANI/PMB dispersions after 60-day storage.

In total, 10 g PVA, 5 g GMA and 90 mL distilled water were introduced into a three-necked flask and stirred at 85 °C until the PVA was completely dissolved, then the pH value was adjusted to 1~2 with HNO_3_ solution. Subsequently, 40 mL CAN solution was dropped into the reaction system in 30 min, and the reaction was kept at 85 °C for 30 min to obtain GMA modified PVA (GPVA). Afterwards, 2 g aniline was added into the GPVA solution while the reaction temperature was kept at 60 °C. After 2 h, the pH value was adjusted to 2 with HCl and the temperature was decreased to 0 °C by using an ice bath followed by the addition of APS solution. The reaction was kept for 24 h to prepare polyaniline grafted GPVA (GPANI). Then, 15 g of BA and MMA with different weight ratio, 5 mL water and 0.06 g SDS were mixed to obtain monomer mixture. The monomer mixture and APS initiator solution were simultaneously added into the GPANI, and the reaction was kept at 80 °C for 6 h to obtain GPANI/polyacrylate (GPANI-PMB) interpenetrating nanocomposite dispersions. The obtained dispersion was dialyzed with deionized water for 24 h to remove the low molecular weight compounds inside the GPANI-PMB dispersions (Figure 1a). The as-prepared samples were designated as GPANI-PMB1, GPANI-PMB2, GPANI-PMB3, GPANI-PMB4 and GPANI-PMB5 when the weight ratio of BA to MMA was 0:100, 25:75, 50:50, 75:25 and 100:0, respectively.

For comparison, GPANI/PMB physical blend was prepared. A total of 2.1 g alkyl alcohol polyoxyethylene ammonium sulfate (D-18), 1.2 g octylphenol polyoxyethylene ether and 90 g deionized water were introduced into a three-necked flask and stirred at 85 °C until the reaction system became homogeneous. Then, 4 g methyl methacrylate (MMA) and 4 g butyl acrylate (BA) were added into the reaction system, followed by a 25 min high-speed stirring. Subsequently, initiator mixture was prepared by mixing 0.5 g ammonium persulphate (APS), 0.5 g natrium bicarbonate (NaHCO_3_) and 30 g deionized water. One third of initiator mixture was added into the aforementioned reaction system to initiate the radical polymerization, and the reaction was kept for 25 min. Afterwards, 36 g MMA, 36 g BA and the residual initiator solution were simultaneously added dropwise into the reaction system in 5 h. The aqueous polyacrylate (PMB) dispersion was thereby obtained after another 45 min reaction at 85 °C. PMB dispersion was then physically blended with the aforementioned GPANI dispersion to obtain PMB/GPANI dispersion on the basis of the same aniline content (6.67%) with GPANI-PMB3 (Figure 1b).

### 2.3. Preparation of GPANI-PMB and GPANI/PMB Nanocomposite Films

The dialyzed GPANI-PMB and GPANI/PMB aqueous dispersions were cast on polytetrafluoroethylene plate and allowed to dry at room temperature to obtain nanocomposite films.

### 2.4. Characterization

The Fourier transform infrared (FTIR) spectra were obtained by the Bruker Vector-22 FTIR Spectrometer with a resolution of 4 cm^−1^. The particle size of the dispersion was analyzed on the Malvern Mastersizer 2000 particle size analyzer. The morphology of colloidal particles in GPANI-PMB and GPANI/PMB dispersions was observed by FEI Tecnai G2 F20 S-TWIN transmission electron microscope (TEM). The surface morphology and elemental distribution were characterized by Hitachi S-4800 scanning electron microscope (SEM) and EDX mapping. The GPANI-PMB and GPANI/PMB film homogeneity was explored by the ultra-depth-of-field three-dimensional microscope of Japan HIROX company. The mechanical properties were measured on an AI-7000-NGD servo material multifunctional high and low temperature control testing machine, and each sample was measured in triplicate. Dynamic mechanical properties such as storage modulus (G′), loss modulus (G′′) and damping factor (tan δ) of the films were determined using a TA Q800 dynamic mechanical thermal analysis (DMTA) instrument. The experiment was run at a fixed frequency of 2 Hz with 6 N initial static force under extension mode. The temperature was ramped from −50 to 145 °C, at a heating rate of 3 °C/min. Electrical conductivities were measured with the standard four-point probe method using a Lattice 2258C probe instrument (Suzhou Jingge Electronics Co., Ltd., Suzhou, China).

## 3. Results and Discussion

### 3.1. Fabrication of Conductive GPANI-PMB Interpenetrating Nanocomposite and GPANI/PMB Physical Blend

In general, in situ polymerization of aniline monomer in polymer matrix was utilized to fabricate PANI/polymer composite [26]. In this work, a facile RIP approach was utilized, i.e., vinyl monomers were introduced into the GPANI dispersion, and then the initiator was introduced to initiate the radical polymerization of vinyl monomers to prepare GPANI-PMB interpenetrating nanocomposite, as shown in Figure 1a. In addition, GPANI was physically blended with PMB to fabricate GPANI/PMB (Figure 1b). TEM images showed that the colloidal particles of GPANI-PMB3 with smaller particle size dispersed in a more homogeneous way in comparison with that of GPANI/PMB (Figure 1c,d). Significant aggregation was observed for GPANI/PMB, and precipitate appeared at the bottom of the sample vial after 60-day storage. This phenomenon indicated that the RIP approach was able to prepare PANI/polymer dispersion with higher stability.

### 3.2. Structural and Morphological Analysis

**Figure 2:**
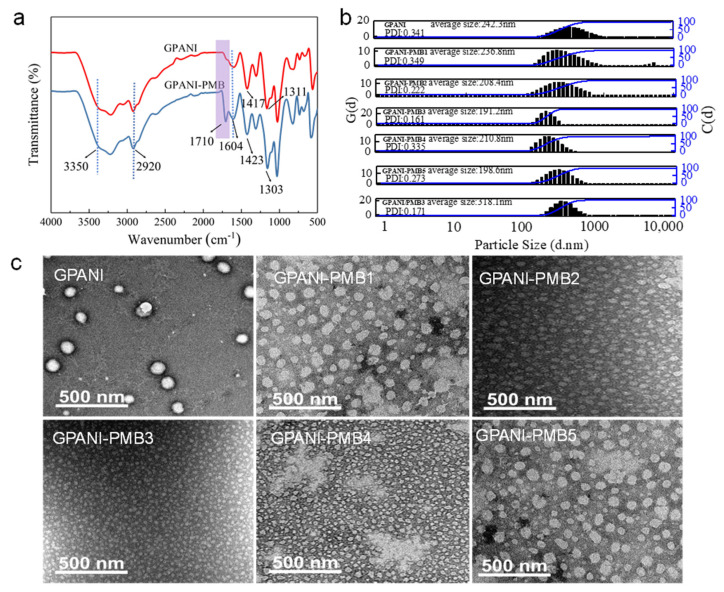
(**a**) FTIR spectra of GPANI and GPANI-PMB3; (**b**) particle size distribution curves and (**c**) TEM images of GPANI, GPANI-PMB1, GPANI-PMB2, GPANI-PMB3, GPANI-PMB4 and GPANI-PMB5.

The structure of GPANI and GPANI-PMB3 were characterized by FTIR (Figure 2a). The peak at 3350 cm^−1^ was caused by the superposition of the stretching vibrations of the -NH group and the residual -OH group. The peak at 2920 cm^−1^ was the symmetry and non-symmetric stretching vibration absorption peak of -CH_2_ [27]. Compared with the FTIR spectrum of GPANI, a new peak at 1710 cm^−1^ appeared in the FTIR spectrum of GPANI-PMB3, which can be assigned to the stretching vibration of C=O, proving the successful incorporation of polyacrylate into the GPANI matrix. The absorption peaks at 1604 and 1417 cm^−1^ were the stretching vibration of the aromatic quinone ring N=Q=N in aniline and the stretching vibration of N-B-N (Q = quinone ring, B = benzene ring) [28]; representative peaks at 1595 and 1423 cm^−1^ corresponded to C=C stretching vibrations of benzenoid and quinoid rings, respectively. Peaks at 1303 and 1311 cm^−1^ were ascribed to the C-N stretching vibration of benzenoid unit. The aforementioned characteristic peaks indicated the presence of PANI structure in GPANI and GPANI-PMB3.

Figure 2b presented the average particle size distributions of GPANI, GPANI-PMB and GPANI/PMB dispersions. The average particle size of the GPANI was 242.3 nm. The particle size of GPANI/PMB increased to 318.1 nm, suggesting that the physical blend of PMB in GPANI increased the size of colloidal particles. In contrast, the particle size of GPANI-PMB prepared by the reverse in situ polymerization of vinyl monomers in GPANI decreased in comparison with GPANI. The particle size of GPANI-PMB3 decreased to 191.2 nm when the weight ratio of BA to MMA was 50:50, followed by a slight increase with continuously increasing the BA content. The decreased particle size suggested that the addition of MMA and BA can promote the dispersion of GPANI colloidal particles and thereby decreased the size of GPANI colloidal particles. Subsequently, the particle size of as-prepared GPANI-PMB dispersion decreased, which can be ascribed to the formation of more uniform GPANI-PMB colloidal particles via the RIP of MMA and BA in GPANI. TEM images (Figure 2c) also demonstrated that the particle size of GPANI-PMB3 dispersion was amongst the smallest and exhibited the most uniform morphology, which was consistent with the particle size results.

### 3.3. SEM and Super Depth-of-Field Microscope Images of Nanocomposite Films

**Figure 3:**
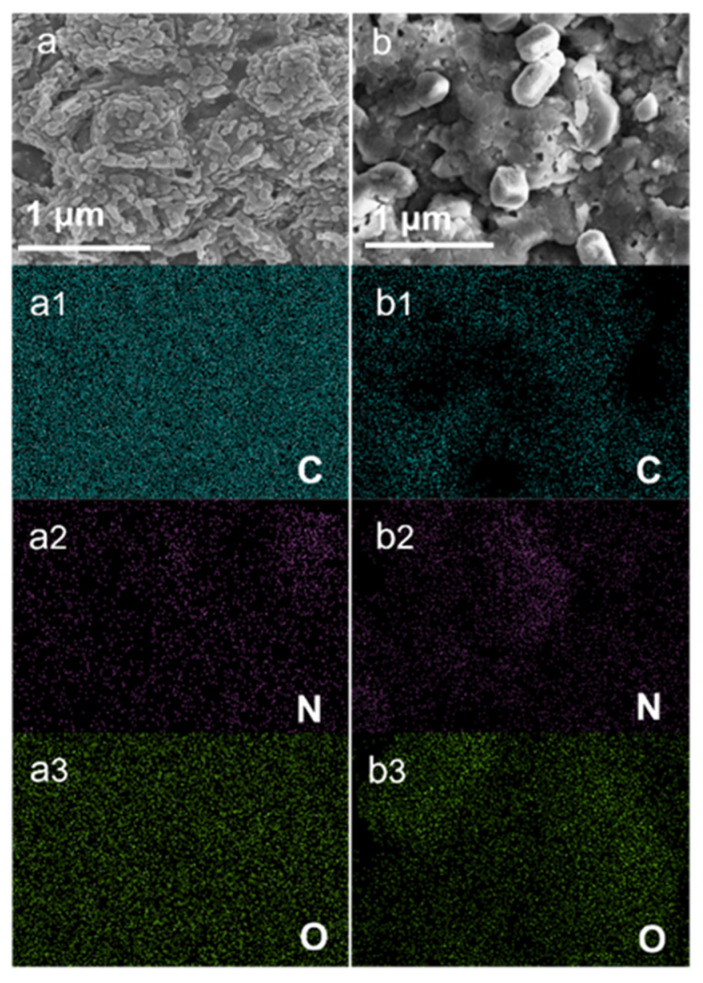
SEM surface images and C, N, O elemental mapping images of (**a**) GPANI-PMB3 and (**b**) GPANI/PMB3 nanocomposite films.

SEM surface morphology and the distribution of GPANI in GPANI-PMB3 and GPANI/PMB3 were investigated by SEM and EDX elemental mapping measurement. GPANI-PMB3 displayed more compact morphology. Smaller GPANI particles dispersed more homogeneously in PMB matrix, presenting pomegranate-shape morphology. EDX elemental mapping images also demonstrated the uniform distribution of C, N, and O elements in GPANI-PMB3 nanocomposite films. In contrast, large-scale aggregation was detected in GPANI/PMB3, resulting in the inhomogeneous elemental distribution, as shown in the Figure 3b. It demonstrated that the reverse in situ polymerization method of vinyl monomer in GPANI was more beneficial to prepare homogeneous PANI/polymer nanocomposite film.

**Figure 4:**
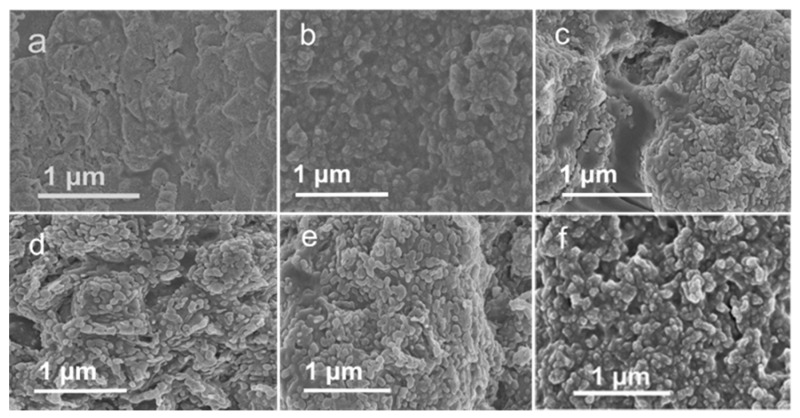
SEM surface images of (**a**) GPANI, (**b**) GPANI-PMB1, (**c**) GPANI-PMB2, (**d**) GPANI-PMB3, (**e**) GPANI-PMB4, and (**f**) GPANI-PMB5 nanocomposite films.

Figure 4 showed the SEM surface morphology of GPANI and GPANI-PMB with different BA/MMA weight ratio. As shown in Figure 4a, GPANI colloidal particles fused together and formed a continuous film. With the incorporation of PMB, the GPANI-PMB became rough, densely distributed with GPANI particles, i.e., the pomegranate-shape morphology became more significant. Additionally, the particle size first decreased and then increased with increasing the BA content. It suggested that incorporating an appropriate ratio of BA and MMA can effectively decrease the particle size and form finer-grained nanocomposite film.

**Figure 5:**
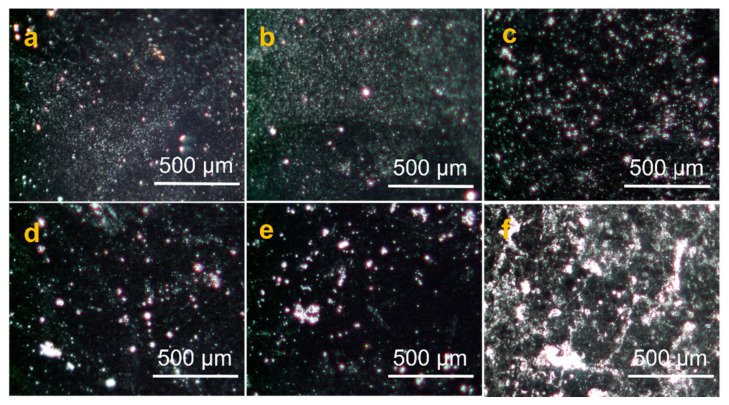
Super depth-of-field microscope images of nanocomposite films: (**a**) GPANI-PMB1, (**b**) GPANI-PMB2, (**c**) GPANI-PMB3, (**d**) GPANI-PMB4, (**e**) GPANI-PMB5 and (**f**) GPANI/PMB3.

In order to further investigate the compatibility and uniformity between GPANI and PMB, super depth-of-field microscope (SDFM) images of GPANI-PMB nanocomposite films with different BA content were provided, as presented in Figure 5. SDFM images also certified that the GPANI-PMB3 nanocomposite film was more uniform compared with other samples. Larger aggregation was observed for GPANI-PMB nanocomposite film when the BA content was higher than 50%. The largest aggregation was observed for GPANI/PMB3, which agreed with the SEM results. SEM, together with SDFM results, proved that the homogeneity of GPANI-PMB nanocomposite film can be significantly improved with incorporating an appropriate amount of BA/MMA. It indicated that the simultaneous incorporation of soft and hard segments can facilitate the uniform distribution of GPANI in PMB, and thereby improved the film homogeneity.

### 3.4. Water Resistance, Mechanical Properties and Electrical Conductivity

**Figure 6:**
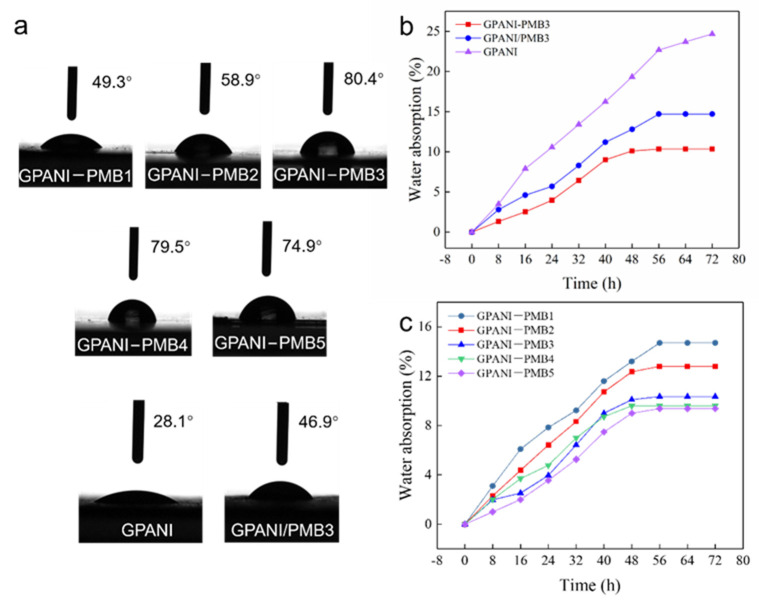
Contact angle (**a**) and water absorption (**b**,**c**) of nanocomposite films.

The contact angle and water absorption of GPANI-PMB and GPANI/PMB nanocomposite films are shown in Figure 6. It was obvious that the contact angle (28.1°) of GPANI significantly increased with the incorporation of PMB, owing to the incorporation of hydrophobic PMB regions. The contact angle increased from 49.3° to 80.4° with increasing the BA content from 0% to 50% (based on the weight of MMA and BA) in GPANI-PMB nanocomposite film, followed by a slight decrease to 74.9°. The contact angle (46.9°) of GPANI/PMB3 was also significantly lower than that (80.4°) of GPANI-PMB3, since the GPANI-PMB3 nanocomposite film was more homogeneous and compact in comparison with GPANI/PMB3.

The variation of water absorption with immersion time for GPANI, GPANI/PMB3 and GPANI-PMB nanocomposite films with different BA/MMA ratio is shown in the Figure 6b,c. As shown in Figure 6b, the water absorption of GPANI and GPANI/PMB3 increased to 24.68% and 14.70% after 72 h immersion, while the water absorption of GPANI-PMB3 only increased to 10.35% with increasing the immersion time, owing to its dense and homogeneous interpenetrating network. In contrast, the water absorption of GPANI-PMB nanocomposite film decreased with increasing the BA content. On the one hand, BA by itself has good hydrophobicity; on the other hand, dense and homogenous interpenetrating network was formed with increasing the BA content, resulting in the increased crosslinking density and water resistance.

**Figure 7:**
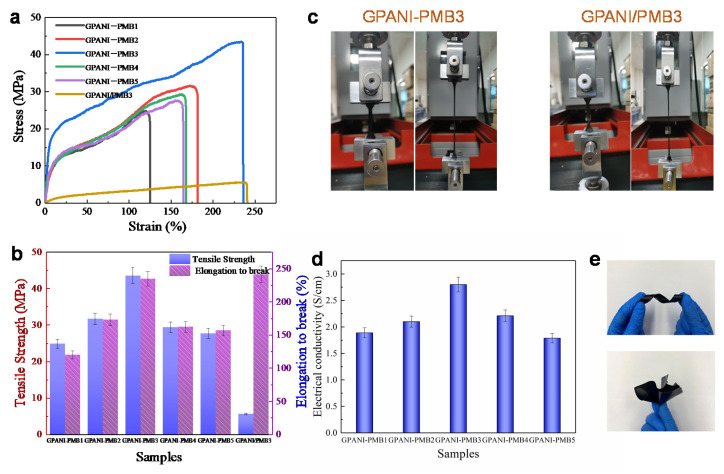
(**a**) Stress–strain curves; (**b**) tensile strength and elongation at break of GPANI-PMB and GPANI/PMB3 nanocomposite films; (**c**) the optical images of GPANI-PMB3 and GPANI/PMB3 nanocomposite films under tensile testing; (**d**) electrical conductivity of the original nanocomposite films without bending; (**e**) the optical images of spiral and flower folds with GPANI-PMB3.

Figure 7a presents the stress–strain curves of GPANI-PMB and GPANI/PMB3 nanocomposite films. The curves showed a strain hardening phenomenon, leading to the increase of strain, and GPANI-PMB transferred from a soft behavior to a ductile behavior in comparison with GPANI/PMB3. Compared with GPANI/PMB3, the tensile strength of GPANI-PMB3 increased from 5.59 to 43.54 MPa, but a slight decrease was observed for the elongation at break. The tensile strength and elongation at break of GPANI were, respectively, 25 MPa and 40%, which were also much lower than that of GPANI-PMB3. These results demonstrated that the formation of uniform interpenetrating PANI/polymer network can significantly improve the mechanical property of nanocomposite films.

The tensile strength of GPANI-PMB increased from 24.82 to 43.54 MPa, and the elongation at break increased from 120.02% to 234.56% with increasing the BA content from 0% to 50%. However, the tensile strength decreased to 27.66 MPa and the elongation at break decreased to 156.70% when the BA content increased to 100%, as illustrated in Figure 7b. The optical images of GPANI-PMB and GPANI/PMB nanocomposite film under tensile testing are shown in Figure 7c, and the folded spiral and flower images of GPANI-PMB3 are presented in Figure 7d, visually certifying the good flexibility of GPANI-PMB3.

**Figure 8:**
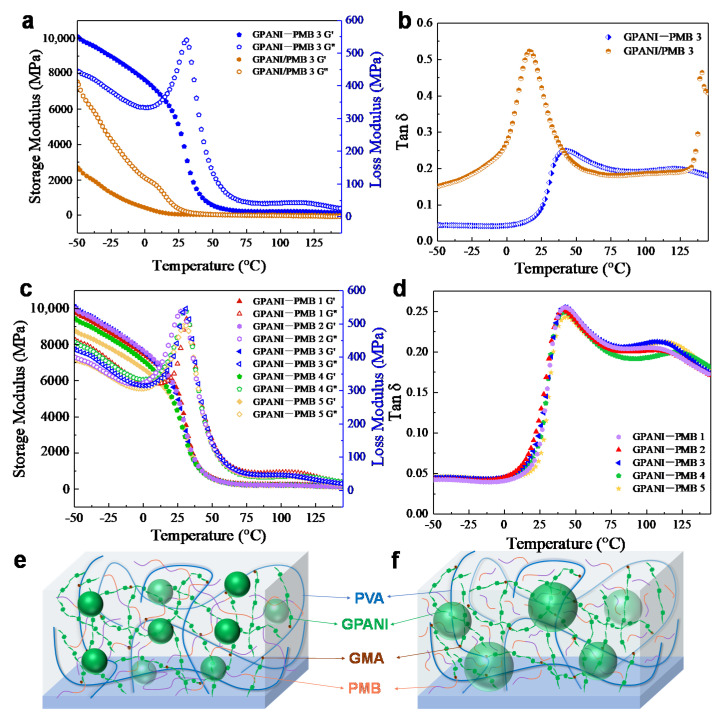
(**a**,**c**) Variation of storage modulus (G′) and loss modulus (G″) with temperature for GPANI-PMB and GPANI/PMB nanocomposite films; (**b**,**d**) tan δ as a function of temperature for GPANI-PMB and GPANI/PMB nanocomposite films; schematic models of (**e**) GPANI-PMB3 and (**f**) GPANI/PMB3 nanocomposite films.

The temperature dependence of the storage modulus (G’), loss modulus (G”) and tan δ for GPANI-PMB3 and GPANI/PMB3 nanocomposite films is shown in Figure 8. In general, G’ represents the rigidity of polymer nanocomposite, and the temperature at the maximum tan δ is regarded as the glass transition temperature [16]. Compared with GPANI/PMB3, the storage modulus of GPANI-PMB3 increased by one order, and the glass transition temperature transferred to the higher temperature, demonstrating the increase of stiffness. In addition, only one relaxation peak was detected in the tan δ-temperature curve of GPANI-PMB3, which two relaxation peaks were observed in the tan δ-temperature curve of GPANI/PMB3. One single relaxation peak implied that the interaction between two pure polymers took place at molecular level, yielding a completely miscible phase [29]. The lowering of tan δ peak intensity was ascribed to the restricted movement of polymer chains owing to the improved interfacial interaction between GPANI and PMB in GPANI-PMB3 [16].

Theoretically, the incorporation of BA can facilitate the mobility of the polymer chains, BA was generally used as “soft monomer” to reduce the minimum film forming temperature, improve the flexibility and toughness of composite film. With respect to GPANI-PMB3, the homogeneous distribution of GPANI in GPANI-PMB nanocomposite film was beneficial for the formation of a more uniform network and enhances molecular interactions more effectively. Moreover, the GPANI particle can functionalize as a nano-reinforcing agent and crosslinking points in the nanocomposite film to enhance the mechanical property. In conclusion, the enhancement in the toughness was due to the synergistic effect of internal antiplasticization and nanoparticle reinforcing function, owing to the formation of uniform GPANI-PMB nanocomposite film (Figure 8e,f).

The electrical conductivity of GPANI-PMB and GPANI/PMB3 nanocomposite films is illustrated in Figure 7d, and the square resistance recorded with the four-probe instrument is shown in Table S1. The electrical conductivity of GPANI with a 13.3% aniline content was 3.21 S/cm, while the electrical conductivity of GPANI/PMB3 was found to be only 0.006 S/cm. In contrast, the electrical conductivity of GPANI-PMB3 reached 2.80 S/cm when the aniline content is only 6.67%, which is generally superior to the similar PANI composites reported in the previous literature (Table S1). It was obvious that only a slight decrease occurred for GPANI-PMB3 when compared with GPANI, further demonstrating the formation of 3D uniform interconnected conductive network. It further demonstrated that RIP method can promote the uniform distribution of PANI in the conventional polymer matrix, and simultaneously improved the water resistance and mechanical properties of nanocomposite polymers.

**Figure 9:**
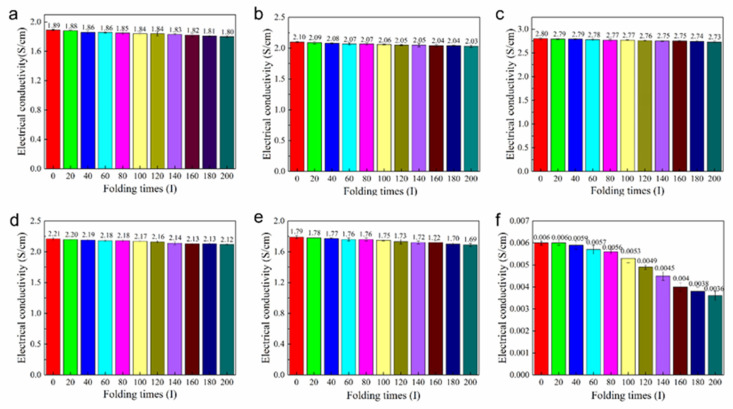
Electrical conductivity of nanocomposite films under different bending conditions: (**a**) GPANI-PMB1, (**b**) GPANI-PMB2, (**c**) GPANI-PMB3, (**d**) GPANI-PMB4, (**e**) GPANI-PMB5, (**f**) GPANI/PMB3 nanocomposite films.

The electrical conductivity after 200-time folding of GPANI-PMB and GPANI/PMB nanocomposite films was also measured, as presented in Figure 9, and the square resistance recorded with the four-probe instrument is shown in Table S1. It was apparent that only a slight decrease took place in the electrical conductivity after folding 200 times, especially for GPANI-PMB3. The electrical conductivity of GPANI-PMB3 only decreased from 2.80 to 2.73 S/cm, while the electrical conductivity of GPANI/PMB3 significantly decreased from 0.006 to 0.0036 S/cm after folding 200 times. It further demonstrated the toughness and stability of 3D conductive network for GPANI-PMB nanocomposite.

## 4. Conclusions

The reverse in situ polymerization method of vinyl monomers in GPANI dispersion was demonstrated to be an effective approach to prepare 3D uniform PANI/polymer interpenetrating nanocomposite with good water resistance, toughness and conductivity. The incorporation of PMB into GPANI can significantly increase the water resistance and toughness. The weight ratio of BA to MMA also played a significant role in the water resistance, mechanical properties and conductivity of GPANI-PMB nanocomposite films. This RIP method should be extensively applicable for preparing high-performance conductive polymer nanocomposites.

## Data Availability

The data presented in this study are available on request from the corresponding author.

## Supplementary Materials

The following are available online at https://www.mdpi.com/article/10.3390/polym13132159/s1, Table S1: Performance comparison for polyaniline composites reported in this work and literatures, Table S2: Resistivity of nanocomposite films measured by four probe technique under different bending conditions.
