# Supplementary material for: Ultraflexible and Mechanically Strong Polymer/Polyaniline Conductive Interpenetrating Nanocomposite via In Situ Polymerization of Vinyl Monomer
Source: Polymers (Basel). 2021 Jun 30;13(13):2159. doi: 10.3390/polym13132159 (PMC8271515; doi:10.3390/polym13132159)
Supplement: Supplementary file 1 [file polymers-13-02159-s001.zip › polymers-1278136-supplementary.pdf]

Supporting information

Ultraflexible and mechanically strong polymer/polyaniline conductive interpenetrating nanocomposite via in situ polymerization of vinyl monomer

Haihua Wang*, Xiaojing Wu, Xuan Qin, Guiqiang Fei*, Liyu Sun, Yanyu Li, Mengxi Wang

Shaanxi Key Laboratory of Chemical Additives for Industry, College of Chemistry and Chemical Engineering, Shaanxi University of Science and Technology, Xi'an, Shaanxi, 710021, PR China

Table S1 Performance comparison for polyaniline composites reported in this work and literatures

Materials	Electrical conductivity(S/cm)	Tensile strength (MPa)	Refs.
Nanostructured polyaniline-clay composite/Polystyrene	0.00076	70.6	[1]
Polyaniline/Polyacrylate	0.10	3.31	[2]
Polyethylene terephthalate/polyaniline	0.76	-	[3]
Polyaniline-clay nanocomposite/3-pentadecyl phenol 4-sulphonic acid	0.63	17.2	[4]
GPANI-PMB	2.8	43.54	This work

Table S2 Resistivity of nanocomposite films measured by four probe technique under different bending conditions

Folding times / Samples	0	20	40	60	80	100	120	140	160	180	200
GPANI-PMB1	0.529	0.532	0.538	0.538	0.541	0.543	0.543	0.546	0.550	0.552	0.555
GPANI-PMB2	0.476	0.478	0.481	0.483	0.483	0.485	0.488	0.488	0.490	0.490	0.493
GPANI-PMB3	0.357	0.358	0.358	0.360	0.361	0.361	0.362	0.364	0.364	0.365	0.366
GPANI-PMB4	0.452	0.455	0.457	0.459	0.459	0.461	0.463	0.467	0.470	0.470	0.472
GPANI-PMB5	0.559	0.562	0.565	0.568	0.568	0.571	0.578	0.581	0.581	0.588	0.592
GPANI/PMB3	166.7	166.7	169.5	175.4	178.6	188.7	204.1	222.2	250	263.2	277.8

References

1 Jds A.; Ss A.; Kp C.; et al. Development of electromagnetic shielding materials from the conductive blends of polystyrene polyaniline-clay nanocomposite. Compos. Part A, Appl. Sci. Manuf. **2010**, 41, 1647-1652.

2 A L W.; A Y G.; A L Z.; et al. Enhanced electrical conductivity and competent mechanical properties of polyaniline/polyacrylate (PANI/PA) composites for antistatic finishing prepared at the aid of polymeric stabilizer. Prog. Org. Coat. **2018**, 125, 99-108.

3 Zhang Y, Pan T, Yang Z. Flexible polyethylene terephthalate/polyaniline composite paper with bending durability and effective electromagnetic shielding performance. Chem. Eng. J. **2020**, 389, 124433.

4 Sudha J.D.; Sivakala S.; Prasanth R.; et al. Development of electromagnetic shielding materials from the conductive blends of polyaniline and polyaniline-clay nanocomposite-EVA: Preparation and properties. Compos. Sci. Technol. **2009**, 69, 358-364.
